# Diagnostic and Prognostic Potential of MicroRNA Maturation Regulators Drosha, AGO1 and AGO2 in Urothelial Carcinomas of the Bladder

**DOI:** 10.3390/ijms19061622

**Published:** 2018-05-31

**Authors:** Anja Rabien, Nadine Ratert, Anica Högner, Andreas Erbersdobler, Klaus Jung, Thorsten H. Ecke, Ergin Kilic

**Affiliations:** 1 Department of Urology, Charité—Universitätsmedizin Berlin, Corporate Member of Freie Universität Berlin, Humboldt-Universität zu Berlin, and Berlin Institute of Health, 10117 Berlin, Germany; n.ratert@gmx.de (N.R.); klaus.jung@charite.de (K.J.); 2 Berlin Institute for Urologic Research, 10117 Berlin, Germany; 3 Institute of Pathology, Charité—Universitätsmedizin Berlin, Corporate Member of Freie Universität Berlin, Humboldt-Universität zu Berlin, and Berlin Institute of Health, 10117 Berlin, Germany; anica.hoegner@charite.de (A.H.); e.kilic@pathologie-leverkusen.de (E.K.); 4 Institute of Pathology, University Medicine Rostock, 18055 Rostock, Germany; andreas.erbersdobler@med.uni-rostock.de; 5 Department of Urology, HELIOS Hospital Bad Saarow, 15526 Bad Saarow, Germany; thorsten.ecke@helios-kliniken.de; 6 Institute of Pathology, Hospital Leverkusen, 51375 Leverkusen, Germany

**Keywords:** bladder cancer, Drosha, AGO1, AGO2, biomarkers, immunohistochemistry

## Abstract

Bladder cancer still requires improvements in diagnosis and prognosis, because many of the cases will recur and/or metastasize with bad outcomes. Despite ongoing research on bladder biomarkers, the clinicopathological impact and diagnostic function of miRNA maturation regulators Drosha and Argonaute proteins AGO1 and AGO2 in urothelial bladder carcinoma remain unclear. Therefore, we conducted immunohistochemical investigations of a tissue microarray composed of 112 urothelial bladder carcinomas from therapy-naïve patients who underwent radical cystectomy or transurethral resection and compared the staining signal with adjacent normal bladder tissue. The correlations of protein expression of Drosha, AGO1 and AGO2 with sex, age, tumor stage, histological grading and overall survival were evaluated in order to identify their diagnostic and prognostic potential in urothelial cancer. Our results show an upregulation of AGO1, AGO2 and Drosha in non-muscle-invasive bladder carcinomas, while there was increased protein expression of only AGO2 in muscle-invasive bladder carcinomas. Moreover, we were able to differentiate between non-muscle-invasive and muscle-invasive bladder carcinoma according to AGO1 and Drosha expression. Finally, despite Drosha being a discriminating factor that can predict the probability of overall survival in the Kaplan–Meier analysis, AGO1 turned out to be independent of all clinicopathological parameters according to Cox regression. In conclusion, we assumed that the miRNA processing factors have clinical relevance as potential diagnostic and prognostic tools for bladder cancer.

## 1. Introduction

In 2012, 430,000 new cases of bladder cancer were diagnosed worldwide. Thus, bladder cancer is the ninth most common cancer in the world with the highest incidence in Northern America, Europe and some countries in Northern Africa and Western Asia [1]. Despite their heterogeneity, several characteristics have been found, which distinguish the subgroups of less aggressive, but often recurring non-muscle-invasive bladder cancer (NMIBC) and the more progressive muscle-invasive bladder cancer (MIBC). The latter holds an overall survival rate of 60% at most [2]. NMIBC develops from urothelial hyperplasia to low-grade carcinoma, with up to 15% proceeding to high-grade tumors. These cancers show characteristic alterations in the Ras-MAPK and PI3K-Akt pathways. The pathway leading to MIBC often includes dysplasia/carcinoma in situ and high-grade non-invasive carcinoma that accumulates common defects, e.g., in tumor suppressors, such as p53 or pRb, or in matrix metalloproteinases [2,3]. Although several potential biomarkers have been tested for their diagnostic and prognostic potential, targets for routine use are still needed.

A recent field of biomarker research has focused on microRNAs (miRNAs), which are involved in various biological processes, including tumorigenesis [4,5]. Several studies profiled the miRNA expression patterns in bladder cancer tissue and indicated some interesting findings regarding its diagnostic and/or prognostic potential [6,7]. The biogenesis of miRNAs is a multistep process involving a couple of protein complexes [8]. In the nucleus, miRNA genes are transcribed by RNA polymerase II/III into a long single or multiple primary miRNA, which is subsequently processed by the “Drosha microprocessor” into hairpin precursor miRNA (pre-miRNA). This “Drosha microprocessor” is a complex of the RNase III enzyme Drosha, its cofactor DiGeorge syndrome critical region gene 8 (DGCR8/Pasha) and other components. The pre miRNA is actively exported by exportin 5/Ras-related nuclear protein-guanosine triphosphate (Ran-GTP) to the cytoplasm. In this cellular compartment, RNase III enzyme Dicer converts pre miRNA to a mature double-stranded miRNA duplex that contains both the mature and its complementary strand and consists of about 20 nucleotides. The mature miRNA is subsequently loaded onto the miRNA Induced Silencing Complex (miRISC). The Argonaute (AGO) family proteins AGO1–AGO4 are the central components of the miRISC complex, which stabilize the mature miRNA strand. The other strand is degraded. AGO2 is the only protein with endonucleolytic activity that mediates the inhibition of target mRNA expression. The subsequent rate of miRNA complementarity and target mRNA affects the repression of translation or cleavage of mRNA. Additionally, there is some evidence indicating an alternative biogenesis pathway in which pre miRNAs are directly loaded onto the miRISC complex after Drosha processing, omitting Dicer processing [9].

As mentioned above, AGO1 and AGO2 are needed for the process of degrading mRNAs or impairing their translation, while Drosha plays a role in initial miRNA maturation. Consequently, it is postulated that they also play a key role in tumor behavior. Some studies have already reported a tumor specific expression of AGO1, AGO2, Dicer and Drosha in the urogenital tract. For example, the Argonautes have been implicated in clear cell renal cell carcinoma [10] and prostate cancer [11]. Meanwhile, the data for Argonautes and Drosha also exist for bladder carcinoma [12,13], but these are contradictory to our findings in major points and require discussion. The aim of our comprehensive immunohistochemical study was to investigate AGO1, AGO2 and Drosha expression in normal bladder urothelium and malignant bladder cancer tissue (NMIBC and MIBC) using a tissue microarray (TMA) as well as to correlate the expression of these proteins with clinicopathological parameters. We believe that our findings will support the potential of these three targets to become bladder carcinoma biomarkers but they will have to be carefully investigated further to avoid inaccurate conclusions indicated by the contradictory results in the literature.

## 2. Results

### 2.1. Immunostaining Pattern of AGO1, AGO2, and Drosha Expression in Bladder Tissue

**Figure 1:**
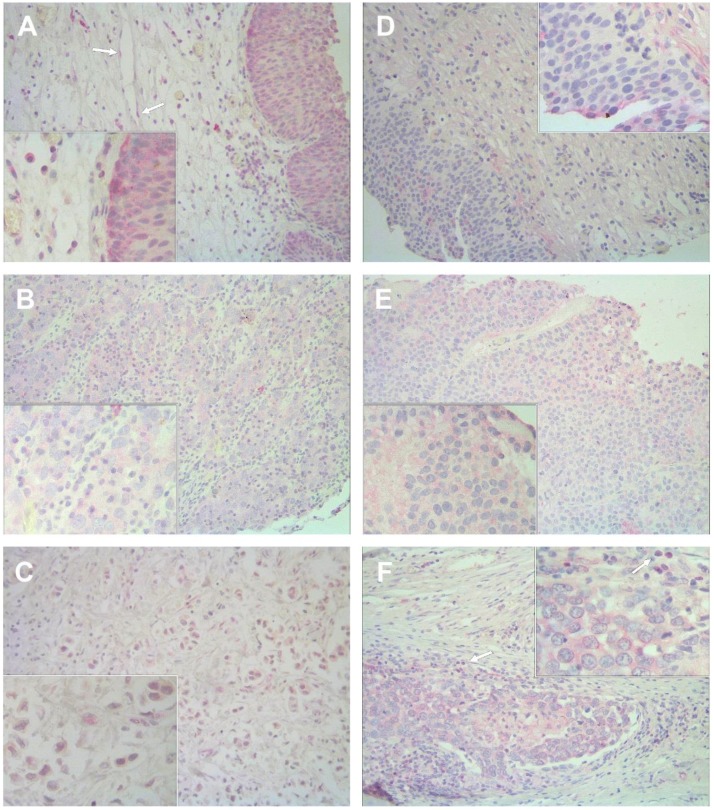
Immunohistochemical staining of AGO1 and AGO2 in bladder tissue. Representative images show the expression of AGO1 in non-malignant bladder tissue (**A**), in non-muscle-invasive pT1 tumor (**B**) and muscle-invasive pT3b tumor tissue (**C**); as well as the expression of AGO2 in normal bladder tissue (**D**), in non-muscle-invasive pTa tumor (**E**) and muscle-invasive pT3b tumor tissue (**F**). The arrows indicate the staining in endothelial cells (**A**) and AGO2 staining of lymphocytes (**F**). Pseudoluminal expression of AGO2 can be seen in (**D**). Magnification: 200×, inserts 400×.

**Figure 2:**
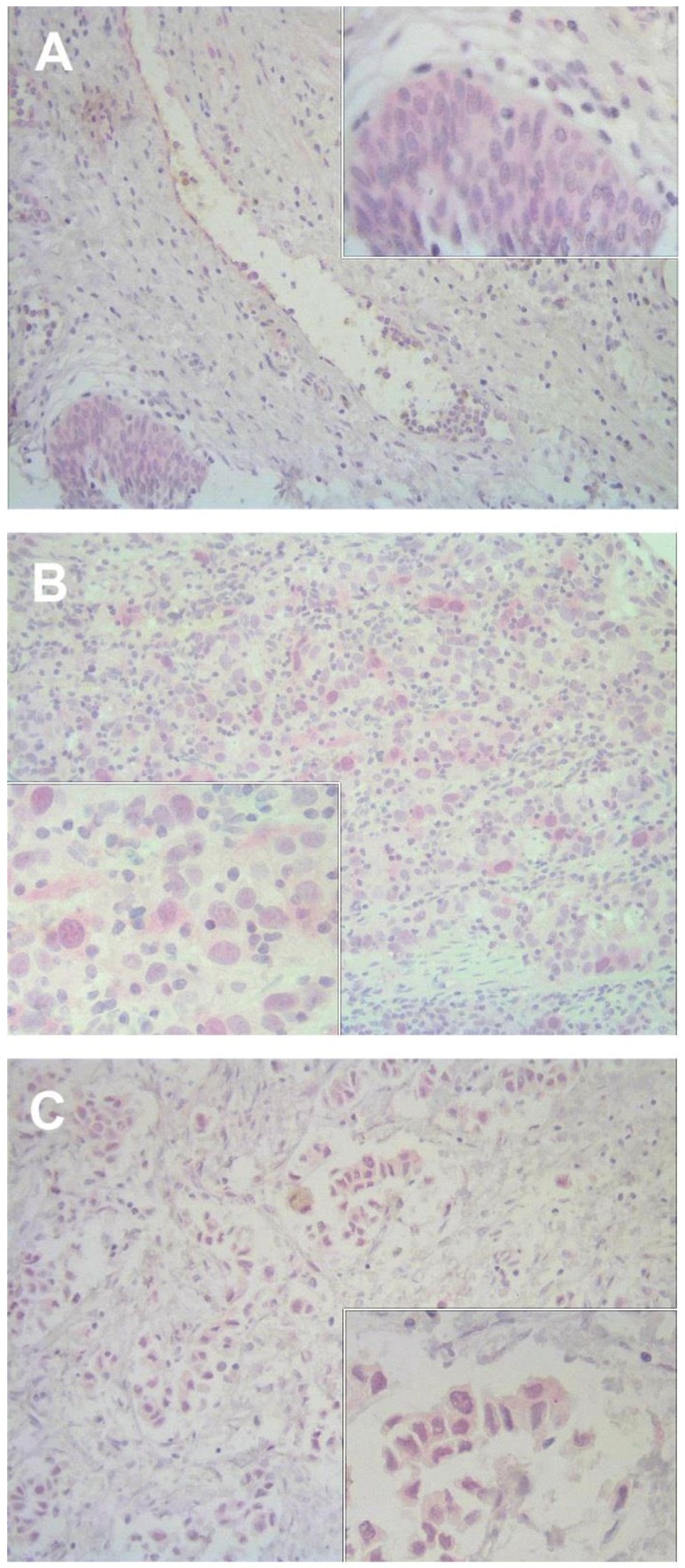
Immunohistochemical staining of Drosha in bladder tissue. Representative images show the expression of Drosha in non-malignant bladder tissue (**A**), in non-muscle-invasive pT1 tumor (**B**) and muscle-invasive pT3b tumor tissue (**C**). The staining of endothelial cells can be seen in (**A**). Magnification: 200×, inserts 400×.

All three targets appeared with a granular pattern in malignant and non-malignant tissue, while they were also often expressed in endothelial cells (Figure 1 and Figure 2). AGO1 staining was found in the cytoplasm and partly in the nuclei of normal and tumor tissue (Figure 1A–C). AGO2 was mainly expressed in the cytoplasm and particularly in the pseudoluminal areas of tumors and adjacent normal tissue (Figure 1D–F). Furthermore, AGO2 was also found in lymphocytes (Figure 1F). Drosha staining was located in the cytoplasm and partly in the nucleus in normal and tumor tissue (Figure 2).

### 2.2. AGO1, AGO2 and Drosha Expression in Bladder Carcinomas Compared to Non-Malignant Tissue and Association with Clinicopathological Parameters

The clinicopathological parameters of the bladder cancer cases are shown in Table 1. AGO1, AGO2 and Drosha were markedly upregulated in NMIBC compared to adjacent normal tissue. However, only AGO2 was significantly upregulated in MIBC (Table 2). Positive AGO2 staining identified 73/109 tumors (67%) without any difference between NMIBC and MIBC when calculating a Fisher’s exact test (Table 3). However, AGO1 and Drosha expression was decreased in NMIBC compared to MIBC (*p* < 0.001, Table 3).

None of the three targets was associated with the clinicopathological parameters of age and sex (Table 3). However, decreased expression of AGO1 (Chi-square test, *p* = 0.001) and Drosha (Chi-square test, *p* < 0.001) was associated with advanced pathological tumor stage and with MIBC compared to NMIBC (Fisher’s exact test, *p* < 0.001) as mentioned above. Drosha levels were also associated with World Health Organization (WHO) grade (Fisher’s exact test, *p* = 0.045). In order to assess the consistency between AGO1, AGO2 and Drosha expression, the McNemar test was utilized. We obtained significant differences between AGO1/AGO2 and AGO1/Drosha expression in bladder tumor tissue samples (*p* < 0.001; Table 3).

### 2.3. Association of AGO1, AGO2 and Drosha Expression with Patient Survival

**Figure 3:**
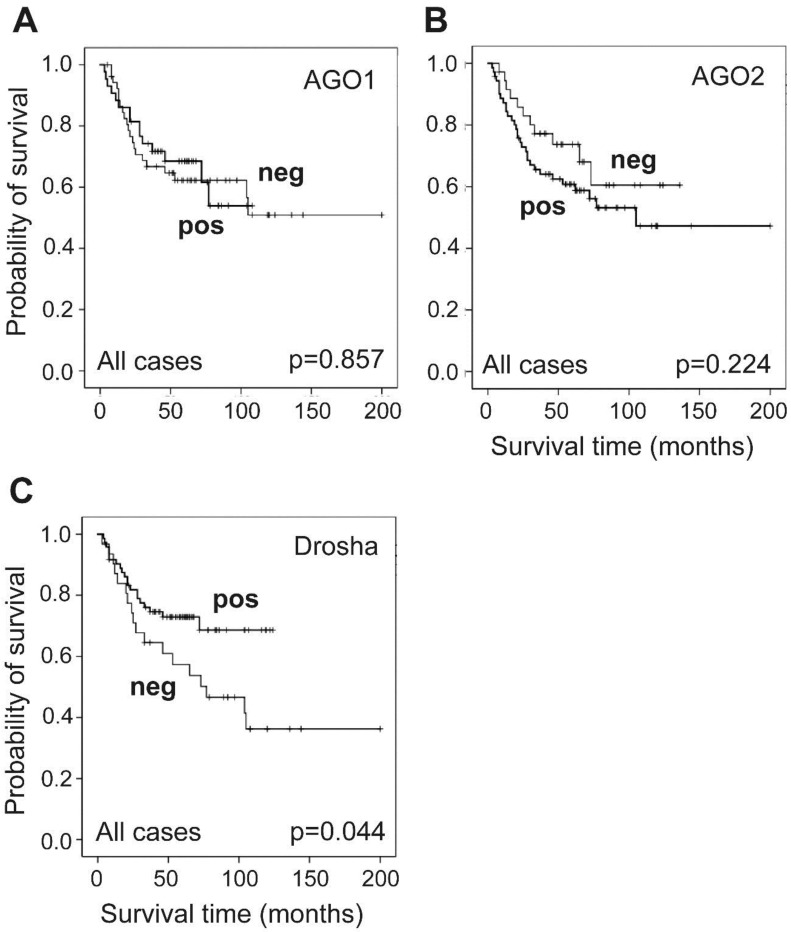
Kaplan–Meier analysis showing overall survival time of bladder cancer patients as a function of AGO1, AGO2 and Drosha levels. Dichotomized expression was not associated with overall survival for 96 cases of AGO1 (**A**) and 107 cases of AGO2 staining (**B**), but Drosha significantly indicated a higher probability of survival with positive expression regarding the 104 available cases (**C**). The overall survival time was defined as the months elapsed between transurethral resection or radical cystectomy and death or the last follow-up date. Censored cases were marked (+). Statistical significance was given as *p* < 0.05. Pos: positive, neg: negative.

The overall survival times available for 110 cases were used in Kaplan–Meier survival analyses, and different subgroups were compared using Chi-square and the log-rank tests. As expected, higher pT stages and tumor grade were significantly associated with reduced patient survival time (*p* < 0.001). We also obtained significant associations by performing separate Kaplan–Meier analyses according to pTa and ≥pT1 tumors (*p* < 0.001) as well as for NMIBC and MIBC (*p* = 0.001). In order to assess the clinical relevance of AGO1, AGO2 and Drosha as prognostic markers in bladder cancer patients, the Kaplan–Meier analyses of dichotomized immunoreactivity data were conducted. AGO1 and AGO2 expression levels that were divided into negative and positive values did not show any significant differences for the available 96 cases and 107 cases, respectively (Figure 3A,B). However, Drosha had a significant correlation with the overall survival time, with a higher probability of survival associated with positive Drosha expression (73 cases, 20 events; 5-year survival of 73%) compared with negative expression (31 cases, 18 events; 5-year survival of 57%) (Figure 3C). Multivariate Cox regression analysis including the clinicopathological parameters of age, sex, pT stage and tumor grade combined with the three targets of AGO1, AGO2 and Drosha (alone or together) did not reveal any statistical significances, while AGO1 turned out to be independent of all patient parameters (Table S1).

## 3. Discussion

Nuclear cleavage of the primary miRNA by Drosha is an essential function in early miRNA maturation. The proteins of the Argonaute family, AGO1 and AGO2, define the next step in miRNA maturation in the cytoplasm and play a key role in post-transcriptional regulation, such as degrading mRNA or impairing its translation. There is an increasing number of studies examining the implication of miRNA maturation regulators in cancer pathobiology, whereas the contribution of Drosha and Argonautes to the diagnosis and clinicopathological behavior of bladder cancer still needs to be clarified. In this study, we investigated the immunohistochemical expression of Drosha, AGO1 and AGO2 proteins in bladder cancer and their association with clinicopathological parameters and overall survival in order to define the diagnostic and prognostic potentials of these miRNA processors for bladder carcinoma.

Higher expression levels of AGO1 and AGO2 associated with tumor progression have been found in different cancer entities, such as in epithelial skin cancer and ovarian cancer [14,15]. There was an upregulation of AGO2 expression in an estrogen receptor α-negative breast cancer cell line, in prostate cancer as well as in esophageal squamous cell carcinoma tissue, which indicates that AGO2 plays a key role in tumorigenesis [16,17]. Furthermore, in hematological cancers, such as multiple myeloma, high AGO2 levels have been reported as a marker of high-risk disease [18]. With regard to bladder carcinoma, previous studies have described the overexpression of Drosha, AGO1 and AGO2 compared to non-malignant bladder tissue [12,13], which we could attribute to NMIBC. In the case of AGO2, this overexpression was found both in NMIBC and MIBC. The differentiation of NMIBC and MIBC cases has not been considered in the earlier studies [12,13].

The association of AGO1 with AGO2 and Drosha according to our McNemar test hints at similar processes and changes of miRNA machinery in bladder cancer, although AGO2 and Drosha were different. With respect to clinicopathological parameters, we found decreased protein expression of AGO1 and Drosha to be significantly associated with higher tumor stage and with MIBC in comparison to NMIBC, although there were no differences for AGO2. In striking contrast, Yang et al. [12] detected a significant association of increased AGO2 levels with higher histological grade, lymph node metastasis and distant metastasis of bladder carcinoma. However, grade and statistical test were not further defined in this study. Zhang et al. [13] claimed that higher AGO2 and Drosha expression was associated with higher histological grade, pT stage (≥T1) and recurrence of bladder carcinoma, although their table data indicated an association with lower grade. These results were inconsistent and the grade was not further defined but we could confirm an association of higher Drosha levels with the lower WHO grade according to criteria of 2016 if applicable to the study above. The decreased expression of AGO1 and Drosha in MIBC compared with NMIBC could suggest a shift from more active miRNA machinery to decreased activity. For bladder cancer, some increased oncogenic miRNAs and many decreased tumor-suppressive miRNAs have been described [19] and thus, a connection could be possible.

Higher Drosha [13] and AGO2 expressions [12,13] have been reported to correspond to shorter overall survival [12] or to shorter recurrence-free and cancer-specific survival [13] in Kaplan–Meier analysis although we only found Drosha to be significantly correlated with prolonged overall survival. Yang et al. [12] contains very few cases in the Kaplan–Meier curves so that the results, especially those indicating that AGO2 is an independent factor, are questionable. However, the other study [13] presents enough cases for sound results and stresses AGO2 as an independent prognostic factor in a reduced model with grade and pT stage only. We could not confirm the prognostic value of AGO2 in our analyses, which had a median observation time of 53 months in contrast to the 36 months [12] and 35 months [13] of the other studies. In contrast, our calculations just proved that AGO1 was independent of clinicopathological parameters, although AGO1 alone in univariate analysis was not of any prognostic value, which was in contrast with Drosha. The significance in the univariate analysis means that the variable can differentiate the lower and higher probability of survival on its own, but the multivariate Cox regression will provide the quality of the variable compared with other (known) patient parameters. According to the Reporting Recommendations for Tumor Marker Prognostic Studies (REMARK), which have been further explained in 2012 [20], we included all variables available in multivariate analysis. Frequently, significant univariate variables lack independence in the multivariate analysis, such as Drosha in our analysis, but there also are variables which only show their quality in multivariate comparison, such as AGO1 in our study. We could confirm the significance of AGO1 in the inclusion model in a backward Likelihood model with the same parameters as in Table S1 (*p* = 0.018). We believe that the value of AGO1 as a prognostic marker should be further evaluated.

Interestingly, in bladder tissue, we found that Drosha was not only present in nuclei, but also in the cytoplasm, which seems contrary to its functional role in nuclear miRNA maturation regulation. However, there are several reports on other tumor entities in which Drosha was observed in both compartments, such as smooth muscle tumors, melanoma, esophageal and breast cancer [21,22,23,24]. The data on localization of Drosha and AGO1 in bladder tissue have been lacking until now because Zhang et al. [13] did not present any in their study. We could confirm mainly the cytoplasmic expression of AGO2 described by Yang et al. [12] but added some details on staining for all three targets, such as the expression in endothelial cells or for AGO2 in lymphocytes, which should be taken into consideration by certain measures, such as Western blot analyses.

As the antibodies used were different from ours (AGO2, [12]), or were not exactly described [13], the difference in the results could have risen from different affinities or specificities, which accentuate the limitations of immunohistochemical studies. Our antibodies were selected according to the literature and were evaluated by an experienced pathologist (E.K.) to avoid artifacts. Another aspect is the difference between our tissue and scoring system (tissue microarray, scoring by intensity) and those of the other groups (full section per case, scoring by intensity and area) [12,13]; both have advantages and disadvantages. For our microarray, we wanted to avoid any bias in an area score for little spots. In histological tissue analysis, the quality of processed formalin-fixed and paraffin-embedded archival material may also influence the staining intensity. In our explorative study, the number of events analyzed was limited because of the limited number of investigated patients for whom comprehensive data were available. This seems to be the main restriction of our work. Further studies are necessary to elucidate the role of Drosha and AGOs in bladder cancer due to the shortcomings of the existing contradictory data.

## 4. Materials and Methods

### 4.1. Tissue Sample Selection

A TMA composed of 112 human urothelial carcinomas of the bladder was used for our retrospective study. Appropriate tissue was selected in accordance to availability and follow-up data of the cases. The study was approved by the Ethics Committee of the HELIOS Hospital in Bad Saarow, Germany (HRC-006913, 21, March, 2012), where all bladder cancer patients underwent radical cystectomy (RTX) or transurethral resection (TUR-B) between 1999 and 2010. Written informed consent was given according to the Declaration of Helsinki. In total, 85 TUR-B and 27 RTX were carried out and none of the patients received any chemotherapy or radiation prior to surgery. For each patient, the following clinical and pathological information was recorded: sex, age, tumor staging according to the International Union Against Cancer, histological grading in accordance with the WHO/ISUP criteria of 2016 and overall survival time in the months after surgery. An overview of patient clinicopathological characteristics is given in Table 1.

The surgical specimens of normal adjacent urothelial and urothelial tumor tissue were fixed in 4% buffered formaldehyde and embedded in paraffin. Histological diagnosis was established on standard hematoxylin and eosin-stained sections by a pathologist. Tumor tissue samples were subdivided into 62 NMIBC (pTa = 42 and pT1 = 20) and 50 MIBC tissue samples (pT2 = 26, pT3 = 18 and pT4 = 6) according to the European Association of Urology guidelines, 2011 [25] (Table 1). Additionally, the adjacent normal bladder tissues of 35 bladder tumor cases without any evidence for reactive histology were included. Patients with carcinoma in situ or metastasis were excluded.

### 4.2. Construction of Tissue Microarray

The areas of bladder carcinoma and adjacent normal tissue were marked on 3-µm hematoxylin/eosin (HE) stained sections of formalin-fixed paraffin-embedded tissue by a pathologist (A.E.) at Charité-Universitätsmedizin Berlin, Germany. Of the corresponding blocks, cores were punched out with a tissue arrayer (1.0 mm diameter; Beecher Instruments, Woodland, CA, USA) according to the previously marked areas, which were subsequently embedded into a new paraffin block as a TMA with maximal 125 cores per block and a tissue of at least 2 mm diameter. Histological conformation was established on HE stained sections (A.E.) according to tumor staging and WHO grading system of 2016.

### 4.3. Immunohistochemistry

Immunostaining was done as described previously [26]. The optimal concentration of the primary antibody was determined in a dilution series on test sections of larger urothelial cancer and corresponding adjacent normal tissue. Finally, the primary antibody against AGO1 (rabbit monoclonal antibody, cat. no. 5053 [clone D84G10]; Cell Signaling Technology, Inc.; Boston, MA, USA) was used at a dilution of 1:50 and incubated overnight at 4 °C. AGO2 (rabbit polyclonal antibody, cat. no. ab32381; Abcam, Cambridge, UK) was used at a dilution of 1:50 and incubated for 1 h at room temperature, while Drosha (rabbit polyclonal antibody, cat. no. ab12286, Abcam) was used at a dilution of 1:250 and incubated for 1 h at room temperature in a humid chamber. Detection was performed by conventional labeled streptavidin-biotin method with alkaline phosphatase as the reporting enzyme. Fast-Red TR/Naphthol AS-MX (cat. no. F4648; Sigma-Aldrich, Munich, Germany) was used as the chromogen. Finally, the TMA was counterstained with hematoxylin and fixed in an aqueous embedding medium. Antibody diluent solution without the application of respective primary antibodies was used as the negative control. Antibodies were checked by Western blotting using the human urinary bladder carcinoma cell lines RT-4 and RT-112 (Figure S1). Immunostainings were evaluated by a pathologist (E.K.) of Charité–Universitätsmedizin Berlin, who was blinded to the clinicopathological data. Immunohistochemical expression of AGO1, AGO2 and Drosha was classified in a binary manner from 0 to 1 according to the following assessment: 0 being no immunoreactivity; and 1 being positive immunoreactivity with staining intensities including cytoplasmic and nuclear staining. The number of assessable cases differed slightly between the three targets, because a few tissue spots disappeared during the staining procedure.

### 4.4. Statistics

Statistical analyses were carried out with SPSS 21.0 (IBM Corp., Somers, NY, USA). Fisher’s exact test, Chi-square test according to Pearson and McNemar test were applied to determine the relationship between AGO1, AGO2 or Drosha immunostaining and clinicopathological characteristics. Univariate analyses for the overall survival time as a function of AGO1, AGO2 or Drosha expression were executed as Kaplan-Meier analyses using the log-rank test. The overall survival time as the primary clinical endpoint was defined as the months elapsed between TUR-B or RTX and either death or the last follow-up date. Multivariate analyses were calculated according to Cox regression. Two-sided *p*-values < 0.05 were considered to be statistically significant in all cases.

## 5. Conclusions

In our study, the altered expressions of AGO1, AGO2, and Drosha were investigated immunohistochemically in bladder cancer to evaluate their diagnostic and prognostic potential in non-invasive and invasive bladder carcinoma. The upregulation of the targets and differentiation of NMIBC and MIBC cases could improve diagnostics; additionally, AGO1 seems to hold prognostic potential for bladder cancer. However, our results stress the need for further research to bridge the gap of prognostic markers in urothelial carcinomas of the bladder. Based on our results, we suggest that Drosha and AGOs are important factors in the tumor biology of bladder cancer.

## Figures and Tables

**Table 1:** Clinicopathological characteristics of the patients undergoing transurethral resection of the bladder or radical cystectomy.

Patient Characteristics (*n* = 112)	*n* (%)
Age, years ^A^	
<69	52 (46.4)
≥69	60 (53.6)
Sex	
female	31 (27.7)
male	81 (72.3)
Tumor characteristics	
pT stage ^B^	
pTa	42 (37.5)
pT1	20 (17.9)
pT2	26 (23.2)
pT3	18 (16.1)
pT4	6 (5.4)
WHO grade ^B^	
low	37 (33.0)
high	75 (67.0)
Operative method	
TUR-B	85 (75.9)
RTX	27 (24.1)
Follow up, months ^C^	
Mean	56
Median	53
Range	3–200
Status after follow-up time ^C^	
alive	67 (60.9)
dead	43 (39.1)

^A^ Age was dichotomized according to median; ^B^ WHO/ISUP criteria of 2016; ^C^ 110 cases available. WHO: World Health Organization; ISUP: International Society of Uropathology; TUR-B: transurethral resection of the bladder; RTX: radical cystectomy.

**Table 2:** Comparison of AGO1, AGO2, and Drosha expression in adjacent normal tissue to NMIBC as well as MIBC in valid cases.

Characteristics	Nonmalignant *n* (%)	NMIBC *n* (%)	MIBC *n* (%)
Argonaute 1	30 (100)	60 (100)	38 (100)
negative	22 (73.3)	23 (38.3)	30 (78.9)
positive	8 (26.7)	37 (61.7)	8 (21.1)
*p* value ^A^		0.003	0.774
Argonaute 2	34 (100)	61 (100)	48 (100)
negative	27 (79.4)	21 (34.4)	15 (31.3)
positive	7 (20.6)	40 (65.6)	33 (68.8)
*p* value ^A^		<0.001	<0.001
Drosha	35 (100)	61 (100)	45 (100)
negative	23 (65.7)	8 (13.1)	23 (51.1)
positive	12 (34.3)	53 (86.9)	22 (48.9)
*p* value ^A^		<0.001	0.255

^A^ Fisher’s exact test. (N)MIBC: (non-)muscle-invasive bladder cancer.

**Table 3:** Immunostaining of AGO1, AGO2 and Drosha associated with clinicopathological parameters of bladder cancer patients.

	AGO1 *n* = 98			AGO2 *n* = 109			Drosha *n* = 106		
Para-meters	neg	pos	*p* value	neg	pos	*p* value	neg	pos	*p* value
*n* (%)	53 (54.1)	45 (45.9)		36 (33.0)	73 (67.0)		31 (29.2)	75 (70.8)	
Age, years ^A^									
<69	23 (23.5)	21 (21.4)	0.839 ^B^	17 (15.6)	33 (30.3)	1.000 ^B^	15 (14.2)	36 (34.0)	1.000 ^B^
≥69	30 (30.6)	24 (24.5)		19 (17.4)	40 (36.7)		16 (15.1)	39 (36.8)	
Sex									
female	14 (14.3)	12 (12.2)	1.000 ^B^	10 (9.2)	20 (18.3)	1.000 ^B^	8 (7.5)	22 (20.8)	0.815 ^B^
male	39 (39.8)	33 (33.7)		26 (23.9)	53 (48.6)		23 (21.7)	53 (50.0)	
pT stage									
pTa	18 (18.4)	23 (23.5)	0.001 ^C^	17 (15.6)	24 (22.0)	0.437 ^C^	3 (2.8)	38 (35.8)	<0.001 ^C^
pT1	5 (5.1)	14 (14.3)		4 (3.7)	16 (14.7)		5 (4.7)	15 (14.2)	
pT2	15 (15.3)	5 (5.1)		9 (8.3)	16 (14.7)		10 (9.4)	13 (12.3)	
pT3	13 (13.3)	1 (1.0)		5 (4.6)	12 (11.0)		12 (11.3)	6 (5.7)	
pT4	2 (2.0)	2 (2.0)		1 (0.9)	5 (4.6)		1 (0.9)	3 (2.8)	
NMIBC	23 (23.5)	37 (37.8)	<0.001 ^B^	21 (19.3)	40 (36.7)	0.838 ^B^	8 (7.5)	53 (50.0)	<0.001 ^B^
MIBC	30 (30.6)	8 (8.2)		15 (13.8)	33 (30.3)		23 (21.7)	22 (20.8)	
WHO grade ^D^									
low	15 (15.3)	20 (20.4)	0.138 ^B^	14 (12.8)	22 (20.2)	0.392 ^B^	6 (5.7)	30 (28.3)	0.045 ^B^
high	38 (38.8)	25 (25.5)		22 (20.2)	51 (46.8)		25 (23.6)	45 (42.5)	
AGO1 ^E^									
neg	-	-	-	21 (21.9)	31 (32.3)	0.025 ^B^	22 (23.7)	27 (29.0)	<0.001 ^B^
pos	-	-	-	8 (8.3)	36 (37.5)		5 (5.4)	39 (41.9)	
AGO2 ^E^									
neg	21 (21.9)	8 (8.3)	<0.001 ^F^	-	-	-	11 (10.7)	23 (22.3)	0.649 ^B^
pos	31 (32.3)	36 (37.5)		-	-	-	19 (18.4)	50 (48.5)	
Drosha ^E^									
neg	22 (23.7)	5 (5.4)	<0.001 ^F^	11 (10.7)	19 (18.4)	0.644 ^F^	-	-	-
pos	27 (29.0)	39 (41.9)		23 (22.3)	50 (48.5)		-	-	-

^A^ Age was dichotomized according to median; ^B^ Fisher’s exact test (*p* < 0.05); ^C^ Chi-square test according to Pearson (*p* < 0.05); ^D^ WHO/ISUP criteria of 2016; ^E^ number of valid cases: Ago1/Ago2 *n* = 96, Ago1/Drosha *n* = 93, Ago2/Drosha *n* = 103; ^F^ McNemar test (*p* < 0.05). AGO: Argonaute; neg: negative, pos: positive staining; (N)MIBC: (non-)muscle-invasive bladder cancer; WHO: World Health Organization; ISUP: International Society of Uropathology.

## Supplementary Materials

Supplementary materials can be found at http://www.mdpi.com/1422-0067/19/6/1622/s1.
