# Supplementary material for: Diagnostic and Prognostic Potential of MicroRNA Maturation Regulators Drosha, AGO1 and AGO2 in Urothelial Carcinomas of the Bladder
Source: Int J Mol Sci. 2018 May 31;19(6):1622. doi: 10.3390/ijms19061622 (PMC6032056; doi:10.3390/ijms19061622)
Supplement: Supplementary file 1 [file ijms-19-01622-s001.zip › Figure S1.pdf]

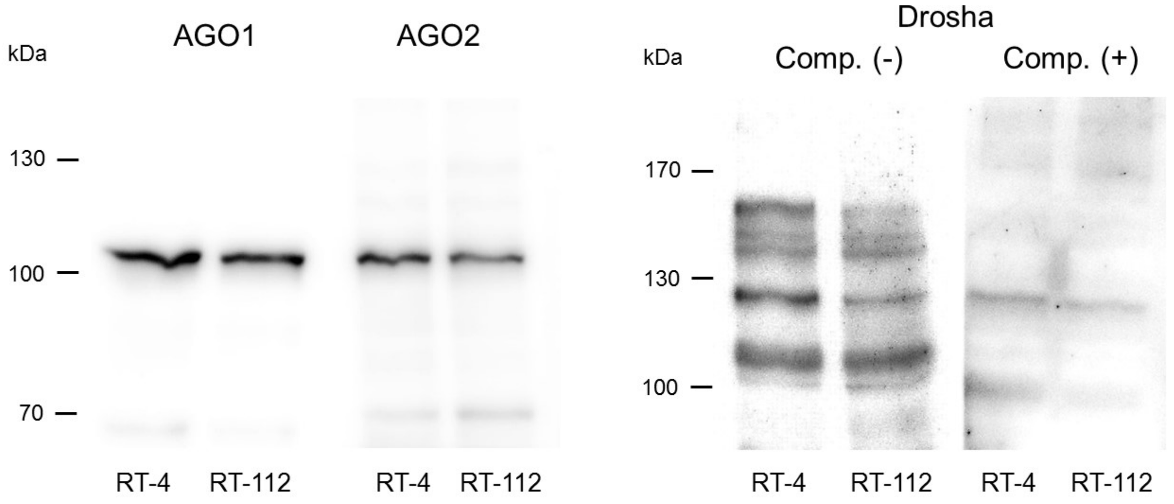

Figure S1. Western Blot analysis of AGO1, AGO2 and Drosha in human urinary bladder carcinoma cell lines RT-4 and RT-112. SDS polyacrylamide gel electrophoresis on a 7% gel (AGOs) or 7.5% gel (Drosha) was conducted using 20 µg protein of cell lysate per lane. Proteins were blotted onto a polyvinylidene fluoride membrane, and unspecific binding was blocked by Roti-Block (Carl Roth, Karlsruhe, Germany) before incubating with the primary antibodies in blocking solution overnight. Antibodies were the same as described for immunohistochemistry in Materials and Methods. For competition, the Drosha antibody was preincubated with ten-fold amount of the appropriate peptide (ab12307, Abcam) to recognize specific bands (Drosha, Comp. (+)). Antibodies were diluted as follows: AGO1 1:1000, AGO2 1:350 and Drosha 1:1000. After incubation with the secondary antibody, a polyclonal goat anti-rabbit antibody coupled to horseradish peroxidase in blocking solution (1:2000, DAKO, Santa Clara, CA, USA), the blots were exposed to Luminata Forte Western HRP Substrate (Millipore/Merck, Darmstadt, Germany) and bands were visualized using an imaging system. The washing buffer for all steps was TBS/0.1% Tween-20. The approximate molecular weight (kDa) was given by the PageRuler Prestained Protein Ladder (Thermo Fisher Scientific, Dreieich, Germany). AGO1 and AGO2 were detected at about 100 kDa confirming the predicted molecular weight of 97 kDa. Drosha isoforms are predicted at 138 kDa and 151–159 kDa, which fits with the bands seen in the range of 130–170 kDa of the marker. Apart from an unspecific band below 130 kDa, which did not disappear in the competition experiment, we saw a band of about 110 kDa that seemed to be specific. A band at about 110–115 kDa was also described in the Abcam website http://www.abcam.com/drosha-antibody-chip-grade-ab12286.html, but there is no explanation, such as one related to Drosha degradation or smaller isoforms, until now. The antibody is widely used for Western Blots and for immunohistochemistry as referenced in the Abcam website. AGO1, AGO2 and the 159 kDa band of Drosha seemed to be decreased in the more aggressive cell line RT-112 compared with RT-4, but evidence would require further experiments.
