# Supplementary material for: Diagnostic and Prognostic Potential of MicroRNA Maturation Regulators Drosha, AGO1 and AGO2 in Urothelial Carcinomas of the Bladder
Source: Int J Mol Sci. 2018 May 31;19(6):1622. doi: 10.3390/ijms19061622 (PMC6032056; doi:10.3390/ijms19061622)
Supplement: Supplementary file 1 [file ijms-19-01622-s001.zip › Table S1.pdf]

Table S1. Univariate and multivariate Cox proportional hazards regression analyses of clinicopathological parameters and immunohistochemical markers AGO1, AGO2 and Drosha for prediction of survival

Variable	Univariate analysis		Multivariate analysis[A]					
	Hazard ratio (95% CI)	P value	Hazard ratio (95% CI)	P value	Hazard ratio (95% CI)	P value	Hazard ratio (95% CI)	P value
**Age, years**[B]								
<69<br>≥69	1.709<br>(0.918-3.182)	0.091	3.510<br>(1.573-7.834)	0.002	1.891<br>(0.993-3.602)	0.053	1.845<br>(0.921-3.693)	0.084
**Sex**								
female<br>male	1.190<br>(0.586-2.416)	0.630	0.902<br>(0.420-1.937)	0.791	1.057<br>(0.516-2.165)	0.880	0.913<br>(0.440-1.896)	0.808
**pT stage**								
pTa/1(NMIBC)<br>pT2-4 (MIBC)	2.903<br>(1.499-5.621)	0.002	4.554<br>(1.774-11.693)	0.002	2.335<br>(1.142-4.775)	0.020	1.877<br>(0.846-4.167)	0.122
**WHO grade**[C]								
low<br>high	5.580<br>(1.990-15.645)	0.001	3.753<br>(1.223-11.515)	0.021	3.793<br>(1.269-11.332)	0.017	3.530<br>(1.172-10.634)	0.025
**AGO1**								
negative<br>positive	0.941<br>(0.481-1.841)	0.858	2.878<br>(1.196-6.923)	0.018	-	-	-	-
**AGO2**								
negative<br>positive	1.526<br>(0.766-3.037)	0.229	-	-	1.420<br>(0.710-2.840)	0.322	-	-
**Drosha**								
negative<br>positive	0.523<br>(0.274-0.996)	0.049	-	-	-	-	0.844<br>(0.419-1.699)	0.634

[A]Inclusion model regarding all clinicopathogical variables; [B]Age was dichotomized according to median; [C]WHO/ISUP criteria of 2016.
Number of valid cases: AGO1 n=96, AGO2 n=107, Drosha n=104, all others (univariate) n=110.
CI: Confidence interval; NMIBC: non-muscle-invasive bladder carcinoma; MIBC: muscle-invasive bladder carcinoma
